# Supplementary material for: Identification of RBPMS as a mammalian smooth muscle master splicing regulator via proximity of its gene with super-enhancers
Source: eLife. 2019 Jul 8;8:e46327. doi: 10.7554/eLife.46327 (PMC6613909; doi:10.7554/eLife.46327)
Supplement: Transparent reporting form [file elife-46327-transrepform.docx]

***eLife’s* transparent reporting form**

We encourage authors to provide detailed information *within their submission* to facilitate the interpretation and replication of experiments. Authors can upload supporting documentation to indicate the use of appropriate reporting guidelines for health-related research (see [EQUATOR Network](http://www.equator-network.org/%20)), life science research (see the [BioSharing Information Resource](https://biosharing.org/)), or the [ARRIVE guidelines](http://www.plosbiology.org/article/info:doi/10.1371/journal.pbio.1000412) for reporting work involving animal research. Where applicable, authors should refer to any relevant reporting standards documents in this form.

If you have any questions, please consult our Journal Policies and/or contact us: [editorial@elifesciences.org](mailto:editorial@elifesciences.org).

**Sample-size estimation**

- You should state whether an appropriate sample size was computed when the study was being designed
- You should state the statistical method of sample size computation and any required assumptions
- If no explicit power analysis was used, you should describe how you decided what sample (replicate) size (number) to use

Please outline where this information can be found within the submission (e.g., sections or figure legends), or explain why this information doesn’t apply to your submission:

According to the ENCODE consortium guidelines for RNAseq experiments (Standards, Guidelines and Best Practices for RNA-Seq, V1.0, June 2011, the ENCODE consortium), at least two biological replicates should be used in RNAseq experiments. Therefore, we decided to use 3 replicates for all the experiments sequenced. Briefly, samples from three biological replicates of RBPMS knockdown in differentiated PAC1 cells and three populations of RBPMS inducible overexpression in proliferative PAC1 cells (from three independent lentiviral transductions) and respective control experiments were sequenced. Replicates sequenced are further explained within the Material and Methods section.

**Replicates**

- You should report how often each experiment was performed
- You should include a definition of biological versus technical replication
- The data obtained should be provided and sufficient information should be provided to indicate the number of independent biological and/or technical replicates
- If you encountered any outliers, you should describe how these were handled
- Criteria for exclusion/inclusion of data should be clearly stated
- High-throughput sequence data should be uploaded before submission, with a private link for reviewers provided (these are available from both GEO and ArrayExpress)

Please outline where this information can be found within the submission (e.g., sections or figure legends), or explain why this information doesn’t apply to your submission:

RNA-Seq data have been deposited as FASTQ files at Gene Expression Omnibus with the reference Series GSE127800:
<https://www.ncbi.nlm.nih.gov/geo/query/acc.cgi?acc=GSE127800>

The separate experiments can be accessed as the Sub Series:

GSE127794 RNAseq analysis of primary differentiated rat aorta tissue
compared to proliferative cultured cells

<https://www.ncbi.nlm.nih.gov/geo/query/acc.cgi?acc=GSE127794>

GSE127799 RBPMS knockdown and overexpression in rat PAC1 pulmonary artery smooth muscle cells (SMCs)

<https://www.ncbi.nlm.nih.gov/geo/query/acc.cgi?acc=GSE127799>

Technical vs biological replicates for RNA-Seq.

For Differentiated PAC1 cells, RBPMS and control siRNAs were transfected into cells on three replicate plates for each condition on the same day.

For RBPMS overexpression, cells from three independent lentiviral transductions were plated on duplicate plates and then treated ± Doxycycline.

For primary rat aorta smooth muscle cells, the three replicates were each derived from pooled aorta from 5 rats. Replicate samples of dispersed single cells, Passage 0 and Passage 9 cells were each derived from separate founder pools of 5 rat aortas.

In all the other experiments, involving transient transfections, experiments were conducted as triplicates with transfection of three separate wells of cells.

This information is found in the figure legends and defined in more details in the Material and Methods.

**Statistical reporting**

- Statistical analysis methods should be described and justified
- Raw data should be presented in figures whenever informative to do so (typically when N per group is less than 10)
- For each experiment, you should identify the statistical tests used, exact values of N, definitions of center, methods of multiple test correction, and dispersion and precision measures (e.g., mean, median, SD, SEM, confidence intervals; and, for the major substantive results, a measure of effect size (e.g., Pearson's r, Cohen's d)
- Report exact p-values wherever possible alongside the summary statistics and 95% confidence intervals. These should be reported for all key questions and not only when the p-value is less than 0.05.

Please outline where this information can be found within the submission (e.g., sections or figure legends), or explain why this information doesn’t apply to your submission:

Statistical analysis methods are reported in the corresponding Material and Methods subsection and also in the figure legends. Data and p values are shown as stated in the figure legends.

(For large datasets, or papers with a very large number of statistical tests, you may upload a single table file with tests, Ns, etc., with reference to sections in the manuscript.)

**Group allocation**

- Indicate how samples were allocated into experimental groups (in the case of clinical studies, please specify allocation to treatment method); if randomization was used, please also state if restricted randomization was applied
- Indicate if masking was used during group allocation, data collection and/or data analysis

Please outline where this information can be found within the submission (e.g., sections or figure legends), or explain why this information doesn’t apply to your submission:

Not applicable.

**Additional data files (“source data”)**

- We encourage you to upload relevant additional data files, such as numerical data that are represented as a graph in a figure, or as a summary table
- Where provided, these should be in the most useful format, and they can be uploaded as “Source data” files linked to a main figure or table
- Include model definition files including the full list of parameters used
- Include code used for data analysis (e.g., R, MatLab)
- Avoid stating that data files are “available upon request”

Please indicate the figures or tables for which source data files have been provided:

We have provided additional Supplementary Data files associated with Figures 1, 2, 5 and Figure 1 Supplement 1.
